# In Vitro Studies on Nanoporous, Nanotubular and Nanosponge-Like Titania Coatings, with the Use of Adipose-Derived Stem Cells

**DOI:** 10.3390/ma13071574

**Published:** 2020-03-29

**Authors:** Michalina Ehlert, Aleksandra Radtke, Tomasz Jędrzejewski, Katarzyna Roszek, Michał Bartmański, Piotr Piszczek

**Affiliations:** 1 Faculty of Chemistry, Nicolaus Copernicus University in Toruń, Gagarina 7, 87-100 Toruń, Poland; m.ehlert@doktorant.umk.pl; 2 Nano-implant Ltd. Gagarina 5/102, 87-100 Toruń, Poland; 3 Faculty of Biological and Veterinary Sciences, Nicolaus Copernicus University in Toruń, Lwowska 1, 87-100 Toruń, Poland; kroszek@umk.pl (K.R.); tomaszj@umk.pl (T.J.); 4 Faculty of Mechanical Engineering, Gdańsk University of Technology, Gabriela Narutowicza 11/12, 80-233 Gdańsk, Poland; michal.bartmanski@pg.edu.pl

**Keywords:** Ti6Al4V alloy, anodic oxidation, adipose-derived mesenchymal stem cells, nanomechanical properties, wettability, biocompatibility

## Abstract

In vitro biological research on a group of amorphous titania coatings of different nanoarchitectures (nanoporous, nanotubular, and nanosponge-like) produced on the surface of Ti6Al4V alloy samples have been carried out, aimed at assessing their ability to interact with adipose-derived mesenchymal stem cells (ADSCs) and affect their activity. The attention has been drawn to the influence of surface coating architecture and its physicochemical properties on the ADSCs proliferation. Moreover, in vitro co-cultures: (1) fibroblasts cell line L929/ADSCs and (2) osteoblasts cell line MG-63/ADSCs on nanoporous, nanotubular and nanosponge-like TiO_2_ coatings have been studied. This allowed for evaluating the impact of the surface properties, especially roughness and wettability, on the creation of the beneficial microenvironment for co-cultures and/or enhancing differentiation potential of stem cells. Obtained results showed that the nanoporous surface is favorable for ADSCs, has great biointegrative properties, and supports the growth of co-cultures with MG-63 osteoblasts and L929 fibroblasts. Additionally, the number of osteoblasts seeded and cultured with ADSCs on TNT5 surface raised after 72-h culture almost twice when compared with the unmodified scaffold and by 30% when compared with MG-63 cells growing alone. The alkaline phosphatase activity of MG-63 osteoblasts co-cultured with ADSCs increased, that indirectly confirmed our assumptions that TNT-modified scaffolds create the osteogenic niche and enhance osteogenic potential of ADSCs.

## 1. Introduction

Titanium and its alloys have been widely used in the construction of implants for complementary bone defects in patients after complicated spinal surgery or limb fractures [1,2,3,4,5]. Although titanium-based implants are typically expected to be longlasting, the lack of full biointegration with the bone for long-term survival often occurs and leads to implant failure. Re-surgery to address such failure involves increased risk, complications, and costs. Therefore, one of the key challenges in bone healing and regeneration is the engineering of an implant, which provides osteointegration with enhanced bioactivity and improves implant-host interactions to reduce biological related implant failure. The success of implants is strongly dependent on the fixation of the implant biomaterial to bone and firm bonding, for optimal function and lastingness [6,7,8,9,10,11]. 

One of the surface modification paths in modern implantology, aiming at the enhancement of bioactivity and osteointegration, is the formation of TiO_2_ based coatings of defined structure, architecture, physicochemical, and mechanical properties, on the surface of titanium-based implants [8,9,12]. The low cost and easy electrochemical production of titanium dioxide nanotubular coatings, possessing beneficial properties, i.e., high surface-area-to-volume ratio, strong oxidizing properties, chemical stability, non-toxicity, good mechanical properties, excellent corrosion resistance, and high biointegration activity, is an especially promising modification method [7,8,13,14,15,16,17]. Due to the similar topography to natural bone, such modified surfaces promote direct contact with bone cells and the formation of apatite, which is the main component of bone tissue [8,18,19]. Homogeneous nanotubular, but also nanoporous and nanosponge-like coatings, on the surface of a titanium/titanium alloy implant can be easily and quickly produced by controlled electrochemical anodization procedure, which has been meticulously optimized and described in our earlier reports [20,21,22,23,24,25,26,27,28,29,30]. 

The effectiveness of osteointegration and healing processes is the consequence of the nanostructured surface of the biomaterial, stimulating osteogenic differentiation of mesenchymal stem cells [31,32,33,34,35,36]. This means that in order to osteointegration process be effective, biomaterials must have a promotional and osteoinductive effect on undifferentiated mesenchymal stem cells in the implant environment. They are the first cells colonizing the surface of the implanted material in vivo [37,38,39]. When an injury occurs and when a biomaterial is implanted, stem cells become activated and recruited to the injury site to support tissue repair. Therefore, biomaterial interactions with stem cells are critical for the long-term success of medical devices. In practice the most widely used source of stem cells is bone marrow and the stromal vascular fraction (SVF) of adipose tissue [40]. The collection procedure of bone marrow is painful and provides a limited amount of multipotent cells [41,42,43], however, the second collection procedure has gained more and more attention. SVF is the heterogeneous mixture, which contains many types of cells: fibroblasts, endothelial cells, adipocytes, blood cells, as well as adipose-derived mesenchymal stem cells (ADSCs)) [36,44,45,46]. The latter ones expand easily in the cell culture and age slowly, having a multipotent differentiation capacity comparable to that of bone marrow stem cells [32,36,47,48,49,50]. Therefore, they are considered as one of the most promising sources of cells in tissue engineering to repair, replace, or regenerate tissues and organs damaged by diseases or injuries. The studies investigated the adhesion, proliferation, and differentiation of ADSCs on titania surfaces are relatively rare so far. Our earlier studies, which were carried out using nanofibrous titania coatings [51], as one of the few, showed that ADSC functionalizing the initially unfavorable surface prepare it for more demanding cells, and make it suitable for the cell growth. Obtained results mobilized us to look again at nanoporous, nanotubular, and nanosponge-like titania systems, which were characterized in details in terms of the structure, morphology and biointegrity with fibroblasts and osteoblasts [20,21,22,23,24,25,26,27,28,29,30], but this time in terms of their predisposition towards adhesion, proliferation, and differentiation of mesenchymal stem cells of adipose origin. In our works, we have also tried to draw attention to the influence of surface coating architecture and its physicochemical properties on the effects mentioned above. Moreover, the aim of our research was to assess the possibility of in vitro co-cultures: (1) fibroblasts cell line L929/ADSC and (2) osteoblasts cell line MG-63/ADSCs on nanoporous, nanotubular, and nanosponge-like TiO_2_ coatings. In this paper we describe conclusions concerning the interaction between the adipose-derived stem cells and produced TNT coatings, enriching the existing knowledge about the biointegrating properties of nanoporous, nanotubular, and nanosponge-like systems produced on the surface of a titanium alloy by its electrochemical oxidation. 

## 2. Materials and Methods 

### 2.1. Preparation of Titania Nanoporous, Nanotubular, and Nanosponge-Like Coatings

TNT coatings were produced based on previously optimized anodic oxidation procedure [20,21,22,24,25], using titanium alloy substrates (Ti6Al4V foil, grade 5, 99.7% purity, 0.20 mm thick (Strem Chemicals, Inc. (Bischheim, France), 6 mm × 60 mm pieces), 0.3% HF solution as a electrolyte, and potentials *U* = 5 V (TNT5), 15 V (TNT15), and 40 V (TNT40). The samples after 20 min of anodization were cleaned in deionized water and acetone using ultrasound, dried in an argon stream, and before biological experiments proceeding, sterilized by autoclaving, according to the previously described procedure [24].

### 2.2. Characterization of Titania Coatings

#### 2.2.1. Morphological and Structural Evaluation 

All samples surface morphologies were observed using a Quanta scanning electron microscope with field emission (SEM, Quanta 3D FEG, Huston, TX, USA). Additionally, the morphology and the structure of TiO_2_ coatings were observed by conventional micrographs with transmission electron microscopy TEM (TEM; Tecnai F20 X-Twin, FEI Company, Portland, OR, USA). In order to check and to confirm the amorphousness of the systems, Raman spectroscopy ((Spectrometer RamanMicro 200 PerkinElmer (PerkinElmer Inc., Waltham, MA, USA) (λ = 785 nm)) has been used. 

#### 2.2.2. The Wettability and Surface Free Energy 

The contact angle of studied samples was analysed with the use of the goniometer (DSA 10 Krüss GmbH, Hamburg, Germany) with drop shape analysis software (ADVANCE), To determine the free surface energy mathematical calculations using the Owens–Wendt method were applied [52]. The contact angle was measured using both polar liquid—distilled water and non-polar liquid—diiodomethane. On the surface of each sample (three samples from each series were examined) 3 µL drops of polar liquid and 4 µL drops of non-polar liquid were applied, then, immediately, the contact angle was measured and the average contact angle value for each series was calculated.

#### 2.2.3. Nanomechanical Properties and Surface Roughness

The hardness, reduced Young’s modulus and adhesion were performed using a nanoindenter (NanoTest Vanatage, Micro Materials, Wrexham, UK). A nanomechanical properties study was carried out using a pyramidal Berkovich diamond indenter with an apex angle equal to 124.4 °. For all tested specimens (Ti6Al4V foil, TNT5-40) 25 independent measurements were performed, with maximum force 50 mN, the loading time 15 s, unloading time 10 s and dwell time with maximum force 5 s. The distance between the indentations was 20 µm. After each measurement a thermal drift correction of 15 s was performed. The Oliver–Pharr method was used to calculate hardness (H) and reduced Young’s modulus (Er). For converting reduced Young’s modulus (Er) to Young’s modulus (E) the Oliver-Pharr methods and a Poisson’s ratio of 0.3 were used [51,53]. To determine the adhesion of surface coatings to the titanium substrate five independent nanoscratch tests on TNT5, TNT15, and TNT40 were performed. The applied force was 0–200 mN with a loading rate of 1.3 mN/s. The distance of nanoscratch-test was 500 µm and distance between nanoscratch-tests was 200 µm. The adhesion of the layers (Lc force) was based on suddenly, abrupt change in friction force (Lf force). Based on information from chart normal force (applied force during tests) in the function of friction forces, the critical force of delamination (Lc) and friction force during delamination (Lf) were determined.

The surface roughness was performing using atomic force microscopy (AFM, NaniteAFM, Nanosurf AG, Liestal, Switzerland) with mode without contact. For all tested specimens (Ti6Al4V foil and TNT5-40) the 512 independent lines were performed on 50×50 µm 50 µm × 50 µm area with force 55 nN. 

### 2.3. Biological Activity Studies of TNT Coatings

#### 2.3.1. Cell Culture 

Adipose-derived human mesenchymal stem cells (ADSCs) were purchased from PromoCell. The culture medium consisted of Mesenchymal Stem Cell Growth Medium® and 10% Supplement Mix® (PromoCell GmbH, Heidelberg, Germany), with 100 IU/mL of penicillin and 100 µg/mL streptomycin (Sigma-Aldrich, Darmstadt, Germany), according to the manufacturer’s protocol. The cells were passaged using 0.04% trypsin-EDTA solution (Sigma-Aldrich) when reaching 70%–80% of confluency.

Human osteoblast-like MG 63 cells (European Collection of Cell Cultures, Salisbury, UK, cat. no. 86051601) were cultured in Eagle’s Minimum Essential Medium supplemented with 2 mM L-glutamine, 1 mM sodium pyruvate, MEM non-essential amino acid, 10% fetal bovine serum (FBS) and 1% antibiotics (penicillin/streptomycin). All components were provided from Sigma Aldrich (Darmstadt, Germany). 

L929 murine fibroblast cells (American Type Culture Collection) were cultured in RPMI 1640 medium supplemented with 2 mM L-glutamine, 10% fetal bovine serum and 1% antibiotics (penicillin/streptomycin). All cell lines were cultured at 37 °C in humidified atmosphere of 5% CO_2_.

#### 2.3.2. Cell Proliferation 

3-(4,5-Dimethyl-2-thiazolyl)-2,5-diphenyl-2H-tetrazolium bromide (MTT) assays were used to evaluate tested specimen’s influence on the cell proliferation after 24 and 72 h. Firstly, we have studied the proliferation level of all three cell lines cultivated on the tested nanocoatings. ADSCs, MG-63 osteoblasts and L929 fibroblasts were seeded onto the autocalvated specimens at a density of 3 × 10^4^, 1 x 10^4^ and 1 × 10^4^ cells, respectively. The cells were cultured for 24 and 72 h. After incubation, 500 µl of MTT (5 mg/mL; Sigma-Aldrich) dissolved in PBS was added to each well, and the plates were incubated in a dark, at 37 °C, humidified atmosphere containing 5% CO_2_ for 3 h. Then, the scaffolds were placed in a new 24-well culture plate, 500 µL of DMSO was added to solubilize the formazan crystals, and aborbance was measured at 540 nm using a microplate reader (Synergy HT; BioTek, Winooski, VT, USA). The specimens incubated without cells was the blank groups. 

In the next experiments, we examined the proliferation level of MG-63 and L929 cells co-cultured with ADSCs on the tested nanolayers. ADSCs were seeded on the specimens at a density of 3 × 10^4^ cells/well and left for 4 h to adhere. Nextly, fibroblasts or osteoblasts at a density of 1 × 10^4^ cells/well were seeded on the attached ADSCs and incubated for 24 and 72 h in a RPMI 1640 or EMEM medium, respectively. After incubation, the MTT assay was performed in the same manner as described above. In the co-culture system, the proliferation level of cells cultured on the Ti6Al4V sample was served as 100%. 

#### 2.3.3. Cell Morphology 

The scanning electron microscopy (SEM; Quanta 3D FEG; Carl Zeiss, Göttingen, Germany) was used to analyse the morphology of ADSCs co-cultured with osteoblasts or fibroblasts growing on the specimens. After 24 or 72 h-lasting incubation, the scaffolds were fixed in 2.5% glutaraldehyde (Sigma Aldrich) followed by they were dehydrated in a graded series of ethanol concentration (50%, 75%, 90%, and 100%). Finally, the samples were dried and the SEM analysis was carried out.

#### 2.3.4. Alkaline Phosphatase Activity

The activity of alkaline phosphatase (ALP) was examined for ADSCs co-cultured with osteoblasts or L929 fibroblasts on the surface of tested nanolayers. After 24 h and 72 h of co-culture under optimal conditions, the cells were lysed with pre-warmed lysis buffer containing 1% TX-100. The obtained samples were centrifuged at 3000× *g*, 5 min, and supernatants (lysates) were collected. To alliquotes of 300 µL sample, the same volume of 4 mM p-nitrophenylphosphate in Tris-HCl buffer pH 9.0 was added. After 60 min incubation the reaction was stopped with 1% NaOH and liberated p-nitrophenol was measured spectrophotometrically at 405 nm. The protein content in each sample was measured by Bradford assay. That allowed calculating the specific activity of ALP.

#### 2.3.5. Statistical Analysis

The values from MTT assays are presented as mean ± SEM. Statistically significant differences among groups were identified by one-way ANOVA with post-hoc Kruskal–Wallis test using GraphPad Prism 7.0 (La Jolla, CA, USA). A p value of less than 0.05 was considered statistically significant.

## 3. Results

### 3.1. TNT Coatings Selected to Biological Experiments; Synthesis, Morphology, and Structure Characterization.

**Figure 1:**
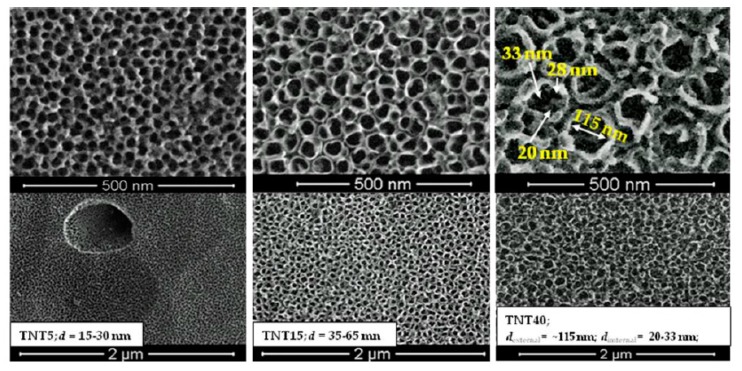
Differences in surface morphology of nanoporous (TNT5), nanotubular (TNT15), and nanosponge-like (TNT40) coatings produced on Ti6Al4V substrates by anodic oxidation method.

Three morphologically different types of TNT coatings formed on the surface of Ti6Al4V alloy substrates in conditions previously reported [20,21,22,24,25], have been selected to biological experiments, i.e. nanoporous (TNT5), nanotubular (TNT15) and nanosponge-like (TNT40) coatings. Scanning electron microscopy (SEM) imaging proved their morphology (Figure 1). Analysis of these data revealed that nanoporous coating (TNT5), of pore diameters 15–30 nm, uniformly covers the entire substrate surface well capturing its shape. The use of higher potentials in the anodization process (*U* = 15, 40V) led to the separation of nanotubes and simultaneously to even the surface of the substrate. The TNT15 and TNT40 coatings consist of tubes of diameter 35–65 nm and 120 ± 10 nm respectively, however, in the last case analysis of SEM images showed the formation of additional, smaller pores and tubes inside greater tubes, resembling nanosponge-like architecture (Figure 1). These three TNT coatings types showed, as expected, an amorphous character, which was confirmed by Raman spectroscopy (Figure S1) and TEM measurements (Figure S2) [54,55,56,57,58]. 

### 3.2. The Wettability and Surface Free Energy 

Data presented in Table 1 revealed that surfaces of TNT5 and TNT15 samples indicated the clear hydrophobic character, as the contact angle (*Θ*) of water in both cases was greater than 90 degrees. The surface of the TNT40 coating showed less hydrophobic character and wettability close to Ti6Al4V sample (Table 1). The free surface energy (SFE) of the produced coatings was calculated using Owens–Wendt method [52]. This method required the contact angles be measured for polar liquid (water) and dispersion liquid (diiodomethane) (Table 1). SFE calculations showed that their values change in the narrow range. As the pore diameter increases, the surface energy firstly increases from 47.8 (mJ/m^2^) for TNT5 up to 61.4 (mJ/m^2^) for TNT15 and then decreases to 47.7 (mJ/m^2^) for TNT40.

### 3.3. Nanomechanical Properties and Surface Topography

**Figure 2:**
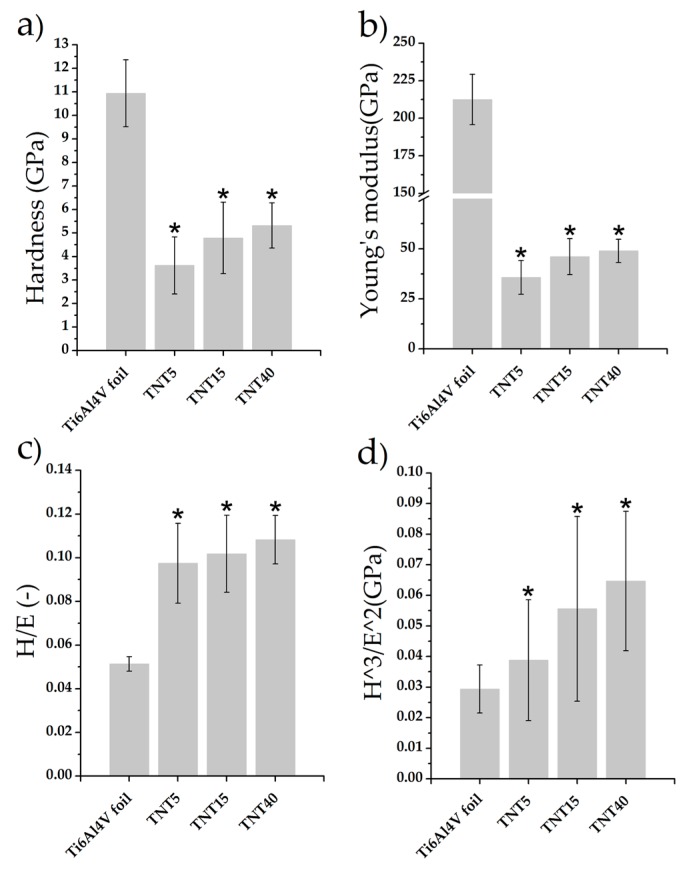
The nanomechanical properties: nanohardness (**a**), Young’s modulus (**b**), H/E ratio (**c**) and H^3^/E^2^ (**d**) of tested reference Ti6Al4V foil, TNT5, TNT15, and TNT40 specimens. Data are presented as the mean ± SD. Asterisks indicate significant differences to the reference specimen Ti6Al4V foil (* *p* < 0.05; ANOVA with Tukey post hoc correction).

The changes in nanomechanical properties of studied coatings, such as hardness, Young’s modulus, and H/E and H^3^/E^2^ coefficients, are shown in Figure 2. The H/E ratio is associated with wear resistant of tested materials and was not tested before. The H^3^/E^2^ ratio can be used to describe of the resistance of the material to plastic deformation, which can be associated with resistant to cracks [59,60]. The highest value of hardness and Young’s modulus were obtained for reference Ti6Al4V foil (10.94 ± 1.42 GPa and 212.48 ± 16.69 GPa, respectively). A significant decrease in both values H and E was found for all the tested modifications. The obtained measurement uncertainties testify to the correctness of the tests performed. Performing 25 measurements using a low value of force on the material with the morphology shown on Figure 1 may associate with obtaining a result with a large standard deviation. The decrease in mechanical properties due to modification of the surface of the Ti6Al4V foil in the case of materials intended for load-bearing implants is a positive phenomenon. The results obtained for the TNT5/TNT15/TNT40 group are similar to each other. The significant difference between hardness and Young’s modulus value for Ti6Al4V foil is the result of low values of determined parameters (0.0513 ± 0.0034 and 0.0294 ± 0.0078 GPa). In the case of coatings TNT5/TNT15/TNT40 an increase in parameters was observed compared to the reference Ti6Al4V foil, which confirms the positive impact of used modifications. 

**Figure 3:**
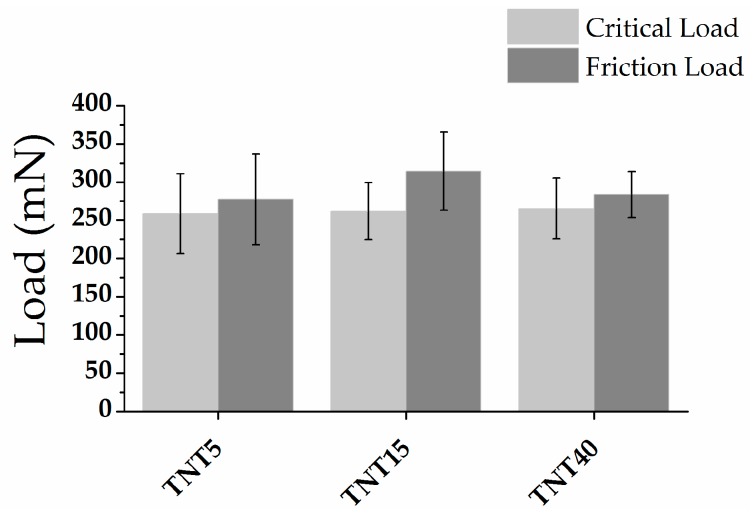
The nanoscratch-test results (critical load and friction load) of tested TNT5, TNT15, and TNT40 specimens.

The nanoscratch-test results were performed on Figure 3. The results correlate with values H/E and H^3^/E^2^ ratios. For studied specimens the adhesion of layers was very similar and did not differ significantly, however, TNT15, for the sake of a well-organized nanotubular architecture, shows the highest adhesion of the tested systems. 

**Figure 4:**
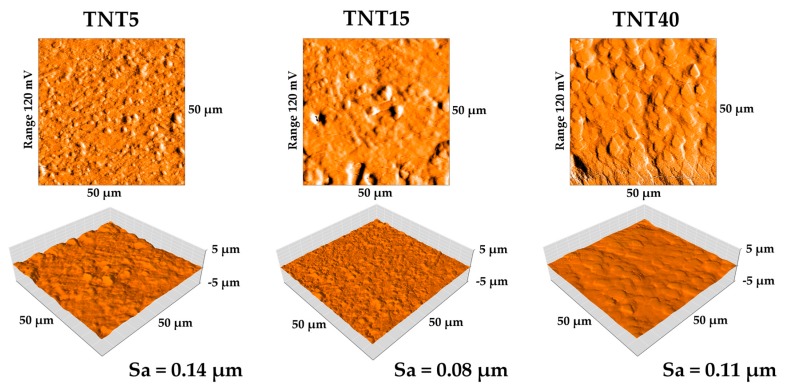
AFM topography of TNT5, TNT15, and TNT40 coatings with Sa parameter values**.**

The surface topography of modified TNT5/TNT15/TNT40 specimens and their surface roughness parameters (Sa) value determined by atomic force microscopy are presented on Figure 4. The surface roughness of specimens after modification (TNT5-40) increased compared with reference Ti6Al4V, for which Sa value has been estimated as 0.03 µm. The results correlate with the results presented in the SEM pictures (Figure 1), where three different morphologies of nanotubes were obtained. The largest increase in roughness relative to the reference sample was observed for the TNT5 sample. An increase in voltage during the oxidation process initially resulted in a decrease in roughness, followed by its increase.

### 3.4. Proliferation Level of Cells Growing on TNT Coatings

**Figure 5:**
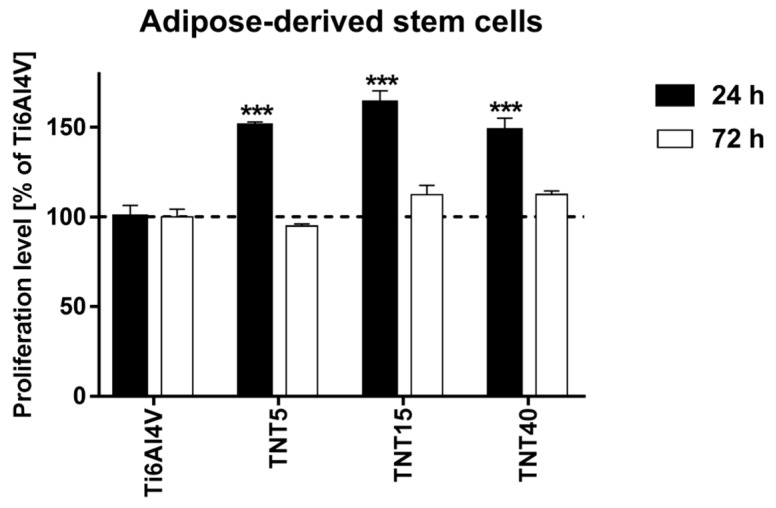
Proliferation level (measured after 24 and 72 h using MTT assays) of adipose-derived mesenchymal stem cells cultured on the TNT coatings compared with the reference Ti6Al4V alloy foils, which was served as 100%. Data are shown as mean ± SEM of four independent experiments. Asterisks indicate differences between the cells growing on the tested specimens in comparison with Ti6Al4V samples. (*** *p* < 0.001). The horizontal line shows the cell proliferation level cultured on the Ti6Al4V.

Cell proliferation level on the TNT coatings, which revealed the differents nanoarchitectures, was evaluated for three cell lines: ADSCs, L929 fibroblasts, and MG-63 osteoblasts after 24 and 72 h of incubation time. All studied TNT coatings proved to be beneficial for adipose-derived stem cells’ viability, in short-term culture. The proliferation level increased by over 50% after 24 h of culture can be explained with accelerated adhesion on the biocompatible surface and resulting higher proliferation level. However, three-day cultures resulted in decreased proliferation or similar to control titanium foils. This may be due to the limitation of the available scaffold surface and the formation of a confluent cell monolayer. It can be also assumed that TNT-modified scaffolds provoked differentiation of ADSCs that is always simultaneous with decreased proliferation level (Figure 5)

**Figure 6:**
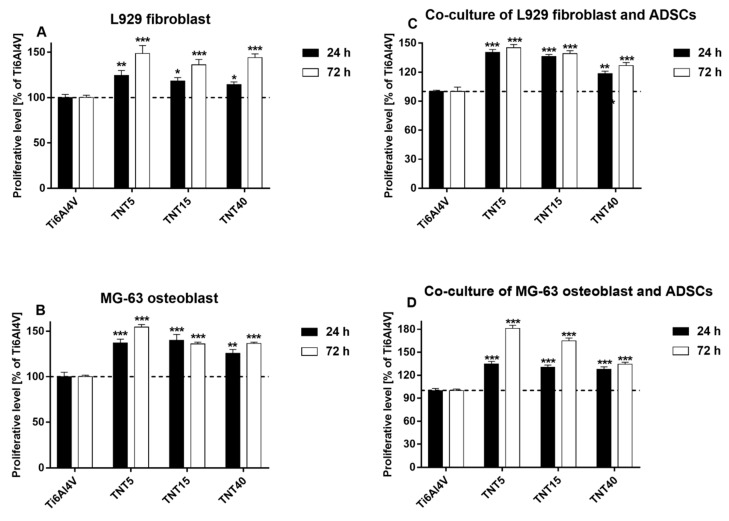
Proliferation level of L929 fibroblasts (**A**) and MG-63 osteoblasts (**B**) cultured on the surface of titania nanoporous (TNT5), nanotubular (TNT15) and nanosponge-like (TNT40) coatings compared with the reference Ti6Al4V samples. Figures (**C**,**D**) show proliferation level of the both cell lines co-cultured with adipose-derived mesenchymal stem cells (ADSCs). Cells were cultivated for 24 and 72 h, and cell viability was measured using MTT assay. The results are expressed as percentage of the cells cultivated (A-B) or co-cultured with ADSCs (C-D) on the reference Ti6Al4V samples (served as 100%). Data are shown as mean ± SEM of four independent experiments. (*** *p* < 0.001, ** *p* < 0.01, * *p* < 0.05, and were considered statistically significant compared to the cells gowing on the Ti6Al4V samples.

All studied TNT coatings provoked also a greater proliferation of L929 fibroblasts as well as MG-63 osteoblasts both after 24 h and 72 h (Figure 6A,B). In the case of L929 fibroblasts, the values measured for the nanolayers were in a range of 114.3%–124.5% after 24 h, and not less than 136% of the reference samples after 72 h. Analysis of MG-63 osteoblasts viability revealed that the proliferation rate of the tested samples was in a range of 125.7%–140.1% after 24 h, and 136.1%–154.6% after 72 h. In the separate study, we measured the proliferation response of fibroblasts and osteoblasts co-cultured with ADSCs on the surface of TNT coatings. As it can be seen in Figure 6C,D, all tested scaffolds induced significant increase in the proliferation rate of L929 fibroblasts (Figure 6C) as well as MG-63 osteoblasts (Figure 6D) co-cultured with ADSCs. This effect was observed both after 24 h and 72 h. The greates cell proliferation level was noticed for TNT5 specimens. For these samples, the viability of L929 fibroblasts co-cultured with ADSCs reached values 140.6 ± 2.9% and 145.3 ± 3.2% after 24 h and 72 h, respectively, while the proliferation level of MG-63 osteoblasts co-cultivated with ADSCs was 134.6 ± 3.5% and 181.0 ± 4.2%, respectively. 

### 3.5. Morphology of Cells Culture on TNT Coatings

**Figure 7:**
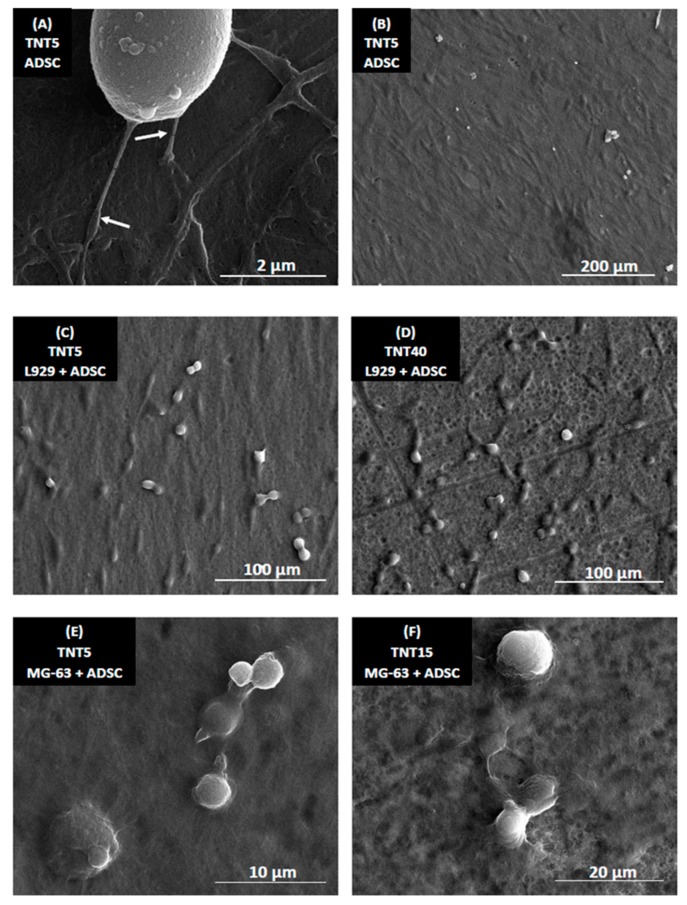
Scanning electron microscopy (SEM) micrographs presenting adipose-derived human mesenchymal stem cells (ADSCs; **A**,**B**), ADSCs co-cultured with L929 fibroblasts (**C**,**D**) or ADSCs co-cultured with MG-63 osteoblasts (**E**,**F**) on the TNT nanocoatings. SEM micrographs present the cells after 72 h of culture. Arrows in image A indicate filopodia, attaching ADSCs to the surface. Specimens’ names and scales of the images were presented in the figures.

Figure 7 presents SEM micrographs of human mesenchymal stem cells (ADSCs; A,B), ADSC co-cultured with L929 fibroblasts (C,D) or ADSC co-cultured with MG-63 osteoblasts (E,F) on the TNT nanocoatings. Regarding the examination by SEM, it was observed that ADSCs formed filopodia (see white arrows in Figure 7A), which effectively attached the cells to the scaffolds surface. Importantly, ADSC had a typical, spindle shape and grew evenly on the entire surface of the nanocoatings (Figure 7B). ADSCs also produced the extracellular matrix when they were cultured on the scaffolds alone or co-cultured with MG-63 osteoblasts. L929 fibroblasts co-cultured with ADSCs also had an elongated shape and they were dividing (Figure 7C,D), indicating their good viability and vitality. The divisions of MG-63 osteoblasts co-cultivated with ADSCs were also observed (Figure 7E,F).

### 3.6. Alkaline Phosphatase Activity

**Figure 8:**
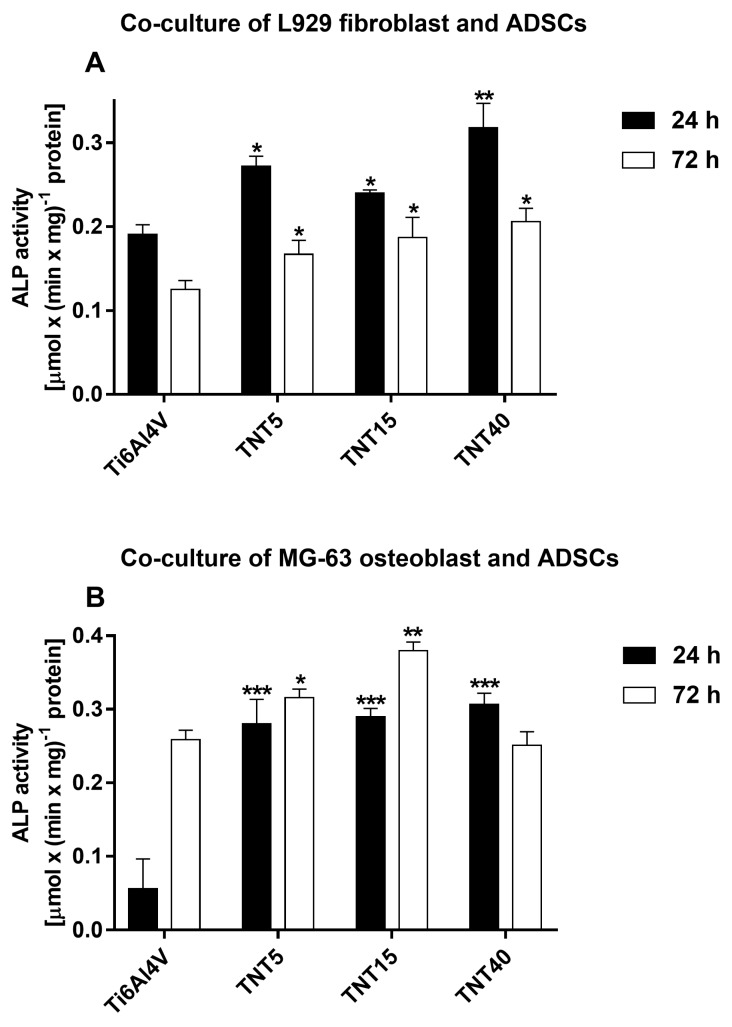
Alkaline Phosphatase (ALP) activity of L929 fibroblasts (**A**) and MG-63 osteoblasts (**B**) co-cultured with adipose-derived mesenchymal stem cells (ADSCs). ALP activity [µmol × (min × mg)^−1^] protein] was expressed as means ± SEM. Asterisks indicate significant differences between the cells growing on the titania nanoporous (TNT5), nanotubular (TNT15) and nanosponge-like (TNT40) coatings compared with Ti6Al4V references sample at the appropriate incubation time (*** *p* < 0.001, ** *p* < 0.01, * *p* < 0.05).

The osteogenic differentiation of ADSCs co-cultures was assessed on the basis of measurement ALP activity normalized to total protein content after 24 and 72 h. Figure 8 presents the comparison of ALP activity measured for fibroblasts or osteoblasts co-cultured with ADSCs on the TNT nanocoatings compared with Ti6Al4V references samples. The ALP activity of L929 cells co-cultured with ADSCs decreased over time (Figure 8A). However, the cells cultivated on TNT coatings still had higher ALP activity than those cultured on reference Ti6Al4V samples. What is more important is that titania nanoporous (TNT5) and nanotubular (TNT15) samples provoked a higher ALP activity over time in MG-63 osteoblasts co-cultured with ADSCs. Particularly, only TNT15 specimens induced the significantly higher ALP activity in osteoblasts co-cultured with ADSCs compared with control samples.

## 4. Discussion

The optimization of stem cells culture conditions and their growth on the implant surface is important issue, which must be taken into account, when titanium or its alloys are surface modified in order to increase their biocompatybility and bioactivity. The chemical surface specificity, e.g., wettability, topography (i.e., pore/tube size, arrangement), roughness, and mechanical properties, are crucial for the characterization of the studied surface and for the estimation of the relationship between the above-mentioned properties and the biocompatybility and bioactivity of the material. According to Roach et al, the surface wettability affects the ability of adhesive proteins to bind to the surface of the substrate, which is associated with the bonding of smaller proteins that reach the surface faster, detaching them and replacing with larger ones [61]. The results of Comelles et al. studies exhibited that the hydrophobicity of the surface may promote cell adhesion in the initial stage, which can be thermodynamically confirmed [62]. According to these reports, in the aqueous environment, the polar groups of proteins are directed outside, while non-polar groups are directed inside the molecule. Near the surface with hydrophobic properties, protein molecules change their conformation by directing their non-polar groups outwards (towards the surface of the substrate). During protein adsorption to the substrate, both the material surface and the protein molecule undergo at least partial dehydration, which occurs most easily, for hydrophobic systems. However, their further proliferation is easier on more hydrophilic surfaces [62]. The data presented in Table 1 and Figure 5 confirms this. The differences in hydrophobicity of studied TNT coatings cause that stem cell adhesion (after 24 h) on TNT15 (*Θ* = 123.3 ± 0.1°) was greater and is reduced for TNT5 (*Θ* = 94.4 ± 0.4°) and TNT40 (*Θ* = 85.3 ± 0.9°). Due to the fact that further division and multiplication of stem cells occurs more easily on more hydrophilic surfaces, after 72 hours we observe a reduction the number of stem cells on the surface of all tested TNT coatings. 

The surface topography is another factor directly influencing the stem cell adhesion and the formation of stable bonds with the implant surface [61]. Topography of the cell influences the ability of the cells to adhere, their morphology, orientation in space, division, and their differentiation. Surface roughness, size, and shape of pores/tubes directly influence the cell adhesion and proliferation [63]. Yang et al. showed that the cells linking only to the surface have contact with a small fragment of the surface, therefore the TNT coatings composed of large diameter tubes (TNT40) are treated by cells as completely smooth similarly, when the pore diameter is very small (TNT5, Figure 1). In such case, the layer of proteins makes it virtually unrecognizable for cells [64]. Surface roughness is closely related to the surface morphology of the substrate. Cell adhesion is more difficult on smoother surfaces due to the smaller actual surface than in the case of rough substrates [65]. In experiments, in which the behavior of osteoblasts and fibroblasts on surfaces with different roughness was compared, it was shown that they prefer substrates with different roughness [66]. According to this research, osteoblasts prefer surfaces with high roughness, their division occurs much faster in such conditions than on smooth surfaces [66]. Contrary, fibroblasts multiply on smooth surfaces [67]. A common effect is a decrease in cell flattening as the surface roughness increases [66,68]. The reason for this phenomenon can be spatial restrictions on rough surfaces, as well as the fact that on smooth surfaces cells need to attach to more points on the substrate to be able to stay adhered [66].

In the case of modifications used for long-term implants, surface roughness is a significant surface parameter. The AFM technique is commonly used to assess surface roughness [60,69,70]. It was found that roughness’ increase had a positive influence on cell proliferation, including osteoblasts [71,72]. It can also determine an increase to bacterial adhesion and proliferation [73], and bacterial infections are one of the main problems of modern implantology [74]. In the presented studies, the modification caused an increase in surface roughness and studies using fibroblasts and osteoblast correlate with the results of AFM causing an increase in cell proliferation with an increase in the Sa parameter. The highest proliferation was obtained for the TNT5 sample, for which the determined roughness was the highest. Antibacteria studies are not the the subject of this publication, however it is worth emphasizing here that in the case of titanium dioxide nanotubes, their natural antibacterial and antifungal properties have been proven and described in our earlier articles [21,22] and in other author’s works [73,75]. Antibacterial additives such as silver were also used to increase properties of titania coatings [27,28,29,30]. 

Regarding the examination by SEM, it was observed that ADSC cells had a typical spindle shape and grew evenly on the entire surface of the nanocoatings. Importantly, ADSCs formed filopodia, which effectively attached the cells to the scaffolds surface despite its hydrophobic nature. It can be concluded that the nanoporous surface is favorable for ADSCs. It should be also noted that bone tissue contains a large number of nanoscale components, therefore, it is beneficial to create an implant surface which imitate this nanoarchitectural hierarchy [76]. ADSCs cells cultured on the scaffolds alone or co-cultured with MG-63 osteoblasts also produced extracellular matrix thus functionalizing the nanocoatings. 

Additionally, MG-63 osteoblasts co-culture with ADSCs indirectly confirmed our assumptions that TNT-modified scaffolds provoked osteogenic differentiation of ADSCs. The total number of osteoblasts seeded and cultured with ADSCs on TNT5 surface raised after 72-h culture almost twice when compared with unmodified scaffold and by 30% when compared with MG-63 cells growing alone. We observe the synergistic effect of improving the surface adhesive properties and creating an osteogenic niche for cultured cells. The results based on MTT assay, were partially confirmed by alkaline phosphatase (ALP) activity measurement. ALP activity is mostly used as a marker of early osteoblast differentation and this enzyme is a byproduct of osteoblast activity, the increased level refering to active bone formation. As can be seen in Figure 8, the ALP activity of L929 cells co-cultured with ADSCs decreased, while activity of MG-63 osteoblasts co-cultured with ADSCs increased over time. Titania nanoporous (TNT5) and nanotubular (TNT15) samples provoked a higher ALP activity and particularly, TNT15 specimens induced the significantly higher ALP activity in comparison with Ti6Al4V alloy foils. More specific molecular tests are required to prove that hypothesis and the direction of differentiation process. We suppose that beside the stimulatory effect on osteoblasts activity, also the limitation of the free available surface and the formation of confluent cell monolayer occur. 

The adhesion of the layer to the titanium substrate is also important factor determining the potential application of modifications on the implant. Forces occurring during the implantation procedure and during subsequent use of the implant in the body can lead to the destruction of poorly adhesive layers. It has been proven that parts of damaged surface modifications can lead to inflammation in the tissues surrounding the implant [77]. Studies on the adhesion of titanium dioxide nanotube layers do not clearly define their adhesive properties. In a study by von Wilmowsky et al. [78] the good adhesion of the nanotubular dioxide layers was confirmed. On the other hand, Kim et al. proved the poor adhesive properties of TiO_2_ [79]. In the presented studies it was proved that the density of packing and porosity of the layer have not a significant influence on the adhesion. Layers with well-organized nanotubular architecture (TNT15) were characterized with the highest adhesion but the differences between the tested specimens were not significant. According the nanoindentation test and nanoscratch-test study specimens TNT15 is the most suitable modification for applications on implant surfaces.

For materials intended for long-term implants, such as titanium alloys, materials should be designed with good mechanical properties but as close as possible to those of surrounding tissues, mainly bones. Numerous studies have confirmed that too much difference between mechanical properties (mainly Young’s modulus) of implant and bone can lead "shielding effect" and consequently bone lose and even loosening of such an implant [80,81,82]. The Young’s modulus of cortical human bone is 10–30 GPa [79]. A positive effect of the modification was observed for all surfaces tested, because their properties are significantly more similar to those of bones. The most similar value of Young’s modulus to bone was obtained for the TNT5 sample (37.88 ± 8.70 GPa), unfortunately this sample also characterized by the lowest nanoindentation properties, wear and cracks resistant which may precludes the use of this modification on long-term implants. The differences in the morphology of the obtained nanotube layer have an impact on nanomechanical properties. Earlier studies have confirmed the impact of nanotube length and width on nanomechanical properties [29]. The presented studies show a clear trend of the impact of voltage increase on the obtained nanomechanical properties. The increase in voltage causes an increase in nanomechanical properties, which is probably associated with a large change in the morphology of the obtained modifications. Coy et al. [59] reported that the value of the H^3^/E^2^ parameter for "hard" coatings should be greater than 0.1, and sometimes even just 0.05. In the present study all tested coating had a higher value.

## 5. Conclusions

The multiannual studies on biomaterials led to the conclusion that during the designing of modern implants, the interactions occurring on the macro-, micro-, and nanoscale on the interface between the living tissue and a biomaterial should be considered. Therefore, besides the bulk physicochemical properties of the material used, which dictate its mechanical and physical properties, the surface properties of the material, especially structure, chemistry (i.e., wettability) and topography must also be taken into account in order to direct a desired cellular response. The expected effects are not always achieved in terms of chemical, biological and mechanical properties. However, optimal properties should be sought, and our research allows us to conclude about the high usefulness of TNT5 coatings. Furthermore, it confirms our extensive previous research, indicating this coating as the most biocompatible, not causing cytotoxic and genotoxic effects and bioactive microbiocidal [20,21,22,23,24,25,26,27,28,29,30,83]. The fact, that TNT5 possessed the most similar value of Young’s modulus to bone, and that on this coating the number of osteoblasts seeded and co-cultured with ADSCs rose after 72 h culture almost twice when compared with unmodified scaffold and by 30% when compared with MG-63 cells growing alone, together with the ability to provoke a higher ALP activity, allows to state that this system has high application potential in modern implantology. 

## Figures and Tables

**Table 1:** Results of contact angle and surface free energy (SFE) measurements; contact angles were measured three times using distilled water and diiodomethane and the SFE was determined using the Owens–Wendt method [52].

Biomaterial Sample	Average Contact Angle (*Θ*) [°] ± Standard Deviation		Surface Free Energy ± Standard Deviation [mJ/m^2^]
	Measuring Liquid		
	Water	Diiodomethane	
Ti6Al4V	81.3 ± 0.2	49.2 ± 0.9	38.5 ± 0.3
TNT5	94.4 ± 0.4	22.4 ± 1.0	47.8 ± 0.3
TNT15	123.3 ± 0.1	31.5 ± 0.7	61.4 ± 0.3
TNT40	85.3 ± 0.9	18.5 ± 0.8	47.6 ± 0.3

## Supplementary Materials

The following are available online at https://www.mdpi.com/1996-1944/13/7/1574/s1, Figure S1: Raman spectra of nanoporous (TNT5), nanotubular (TNT15), and nanosponge-like (TNT40) titania coatings (A—anatase, R—rutile), Figure S2. TEM image (a), HRTEM image (b) and the SAED patterns of TNT15 (c).
