# Supplementary material for: In Vitro Studies on Nanoporous, Nanotubular and Nanosponge-Like Titania Coatings, with the Use of Adipose-Derived Stem Cells
Source: Materials (Basel). 2020 Mar 29;13(7):1574. doi: 10.3390/ma13071574 (PMC7177883; doi:10.3390/ma13071574)
Supplement: Supplementary file 1 [file materials-13-01574-s001.pdf]

Supplementary

In Vitro Studies on Nanoporous, Nanotubular and Nanosponge-Like Titania Coatings, with the Use of Adipose-Derived Stem Cells

Michalina Ehlert [1,2], Aleksandra Radtke[1,2,*], Tomasz Jędrzejewski [3], Katarzyna Roszek [3], Michał Bartmański [4] and Piotr Piszczek [1,2,*]

[1] Faculty of Chemistry, Nicolaus Copernicus University in Toruń, Gagarina 7, 87-100 Toruń, Poland; m.ehlert@doktorant.umk.pl
[2] Nano-implant Ltd. Gagarina 5/102, 87-100 Toruń, Poland
[3] Faculty of Biological and Veterinary Sciences, Nicolaus Copernicus University in Toruń, Lwowska 1, 87-100 Toruń, Poland; kroszek@umk.pl (K.R.); tomaszj@umk.pl (T.J.)
[4] Faculty of Mechanical Engineering, Gdańsk University of Technology, Gabriela Narutowicza 11/12, 80-233 Gdańsk, Poland; michal.bartmanski@pg.edu.pl
* Correspondence: aradtke@umk.pl (A.R.); piszczek@umk.pl (P.P.); Tel.: (+48)600321294 (A.R.); Tel.: +48607883357 (P.P.)

Received: 17 February 2020; Accepted: 26 March 2020; Published: 29 March 2020

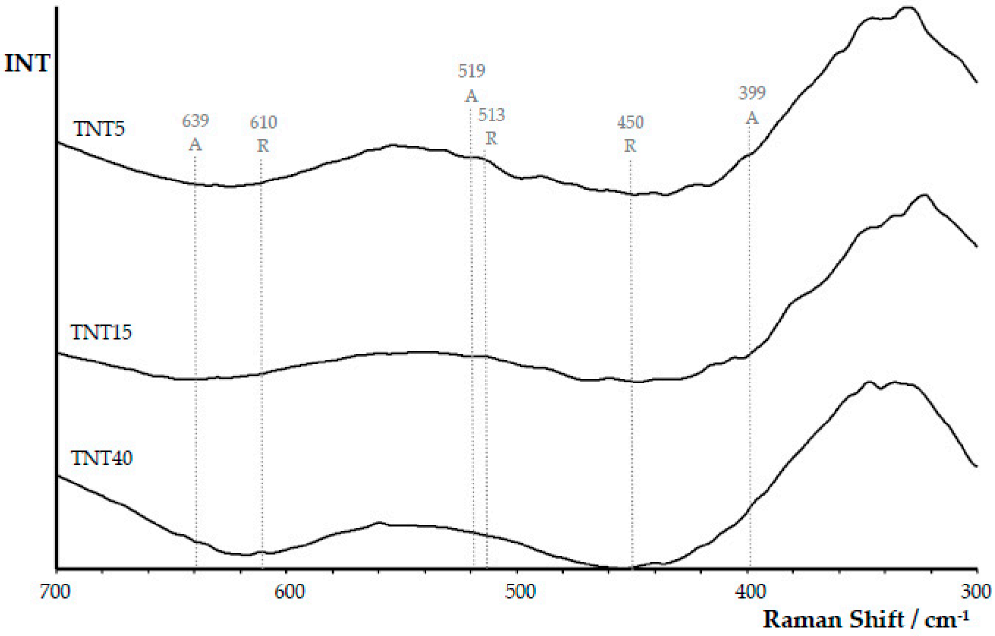

Figure S1. Raman spectra of nanoporous (TNT5), nanotubular (TNT15), and nanosponge-like (TNT40) titania coatings (A—anatase, R—rutile).

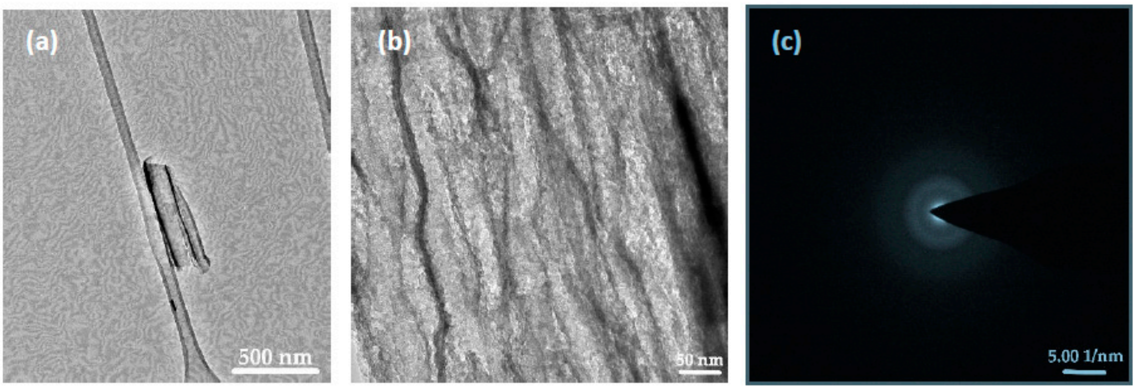

Figure S2. TEM image (**a**), HRTEM image (**b**) and the SAED patterns of TNT15 (**c**).

© 2019 by the authors. Submitted for possible open access publication under the terms and conditions of the Creative Commons Attribution (CC BY) license (http://creativecommons.org/licenses/by/4.0/).
